# The *Arabidopsis thaliana* gene *AtERF019* negatively regulates plant resistance to *Phytophthora parasitica* by suppressing PAMP‐triggered immunity

**DOI:** 10.1111/mpp.12971

**Published:** 2020-07-28

**Authors:** Wenqin Lu, Fengyan Deng, Jinbu Jia, Xiaokang Chen, Jinfang Li, Qujiang Wen, Tingting Li, Yuling Meng, Weixing Shan

**Affiliations:** ^1^ State Key Laboratory of Crop Stress Biology for Arid Areas and College of Plant Protection Northwest A&F University Yangling China; ^2^ State Key Laboratory of Crop Stress Biology for Arid Areas and College of Life Sciences Northwest A&F University Yangling China; ^3^ Institute of Plant and Food Science Department of Biology Southern University of Science and Technology Shenzhen China; ^4^ State Key Laboratory of Crop Stress Biology for Arid Areas and College of Horticulture Northwest A&F University Yangling China; ^5^ State Key Laboratory of Crop Stress Biology for Arid Areas and College of Agronomy Northwest A&F University Yangling China

**Keywords:** *Arabidopsis thaliana*, oomycete, *Phytophthora parasitica*, susceptibility, transcription factor

## Abstract

*Phytophthora* species are destructive plant pathogens that cause significant crop losses worldwide. To understand plant susceptibility to oomycete pathogens and to explore novel disease resistance strategies, we employed the *Arabidopsis thaliana–Phytophthora parasitica* model pathosystem and screened for *A. thaliana* T‐DNA insertion mutant lines resistant to *P. parasitica*. This led to the identification of the resistant mutant 267‐31, which carries two T‐DNA insertion sites in the promoter region of the *ethylene‐responsive factor 19* gene (*ERF019*). Quantitative reverse transcription PCR (RT‐qPCR) assays showed that the expression of *ERF019* was induced during *P. parasitica* infection in the wild type, which was suppressed in the 267‐31 mutant. Additional *erf019* mutants were generated using CRISPR/Cas9 technology and were confirmed to have increased resistance to *P. parasitica*. In contrast, *ERF019* overexpression lines were more susceptible. Transient overexpression assays in *Nicotiana benthamiana* showed that the nuclear localization of *ERF019* is crucial for its susceptible function. RT‐qPCR analyses showed that the expression of marker genes for multiple defence pathways was significantly up‐regulated in the mutant compared with the wild type during infection. Flg22‐induced hydrogen peroxide accumulation and reactive oxygen species burst were impaired in *ERF019* overexpression lines, and flg22‐induced MAPK activation was enhanced in *erf019* mutants. Moreover, transient overexpression of *ERF019* strongly suppressed INF‐triggered cell death in *N. benthamiana*. These results reveal the importance of *ERF019* in mediating plant susceptibility to *P. parasitica* through suppression of pathogen‐associated molecular pattern‐triggered immunity.

## Graphical abstract

*ERF019* is induced during *Phytophthora parasitica* infection and acts as a negative regulator of plant resistance by suppressing PAMP‐triggered immunity.

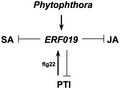

## 1 INTRODUCTION

The “plant destroyer” *Phytophthora* causes devastating disease in a large number of crops and forest seedlings worldwide. For example, potato late blight caused by *Phytophthora infestans* can lead to severe decreases in production and also serious economic losses (Haverkort *et al*., 2008). *P. sojae*, *P. ramorum*, *P. parasitica*, and *P. capsici* can also cause important agricultural diseases such as soybean root rot and oak stagnation (Tyler, 2002; Grünwald *et al*., 2012; Lamour *et al*., 2012; Meng *et al*., 2014; Kamoun *et al*., 2015; Panabières *et al*., 2016).

Plants have sufficient weapons to repel pathogen attacks, but need to recognize the pathogen in time, which mainly occurs through two different systems. One is referred to as PAMP‐triggered immunity (PTI), which is activated by transmembrane pattern‐recognition receptors (PRRs) through recognition of pathogen‐ or microbe‐associated molecular patterns (PAMPs or MAMPs) (Jones and Dangl, 2006; Boller and Felix, 2009; Bigeard *et al*., 2015; Boutrot and Zipfel, 2017) and initiates a series of immune responses, including reactive oxygen species (ROS) bursts, callose deposition, biosynthesis of phytohormones (such as salicylic acid [SA], jasmonate [JA], and ethylene [ET]), and the expression of a large number of defence‐related genes (Zipfel *et al*., 2004; Naito *et al*., 2008). The other system is called effector‐triggered immunity (ETI), which is based on the specific recognition between pathogen effectors and plant resistance (R) proteins, according to the gene‐for‐gene theory. This recognition leads to a rapid and localized hypersensitive response (HR, cell death) at infection sites and inhibits pathogen colonization (Jones and Dangl, 2006).

Although priming of immunity responses when pathogens attack is the key to resistance, excessive and inappropriate defence responses interfere with the growth of a plant. To mitigate the trade‐off between growth and defence, plants have evolved a series of mechanisms to negatively regulate defence pathways. For example, the rice *Pigm* locus confers durable resistance to the fungus *Magnaporthe oryzae* without yield penalty; this is achieved through epigenetic regulation of two antagonistic receptors, PigmR and PigmS, encoded by this locus (Deng *et al*., 2017). PigmR confers broad‐spectrum resistance, whereas PigmS competitively attenuates PigmR homodimerization to suppress resistance (Deng *et al*., 2017). In addition, growth‐related hormones, auxin, brassinosteroids (BRs), and gibberellins (GAs) can directly or indirectly negatively regulate PTI‐mediated defence (Yamada, 1993; Chen *et al*., 2007; Albrecht *et al*., 2012; Jaillais and Vert, 2012). However, these negative regulatory pathways can be hijacked by effectors secreted by pathogens to promote infection (Jones and Dangl, 2006). For example, plant cinnamyl alcohol dehydrogenase 7 (CAD7), which is involved in the negative regulation of plant resistance to *Phytophthora* pathogens including *P. infestans*, *P. paratisica*, and *P. capsici*, is a common target of multiple AVR3a‐like effectors from *Phytophthora* pathogens. These effectors suppress PTI responses by stabilizing CAD7 (Li *et al*., 2019). In potato, StVIK and StKRBP1 are targeted by the RXLR effectors Pi17316 and Pi04089 from *P. infestans*, respectively, to facilitate invasion (Wang *et al*., 2015; Murphy *et al*., 2018). Therefore, appropriate manipulation of negative regulators of plant immunity has the potential to improve broad‐spectrum disease resistance.

To explore the mechanisms by which negative regulators suppress plant resistance to pathogens, we used a model compatible system between *Arabidopsis thaliana* and the oomycete pathogen *P. parasitica* (Wang *et al*., 2011b) to screen for *A. thaliana* T‐DNA insertion mutants resistant to *P. parasitica* infection. We identified an *erf019* mutant that showed less susceptibility to *P. parasitica*. Our analysis revealed that *ERF019* negatively regulates plant defence responses to *Phytophthora* pathogens by suppressing PAMP‐triggered immunity, thus acting as an important regulator in balancing plant disease resistance and growth.

## 2 RESULTS

### 2.1 Identification of an *erf019* mutant, 267‐31, that limits colonization of *P. parasitica*

**Figure 1:**
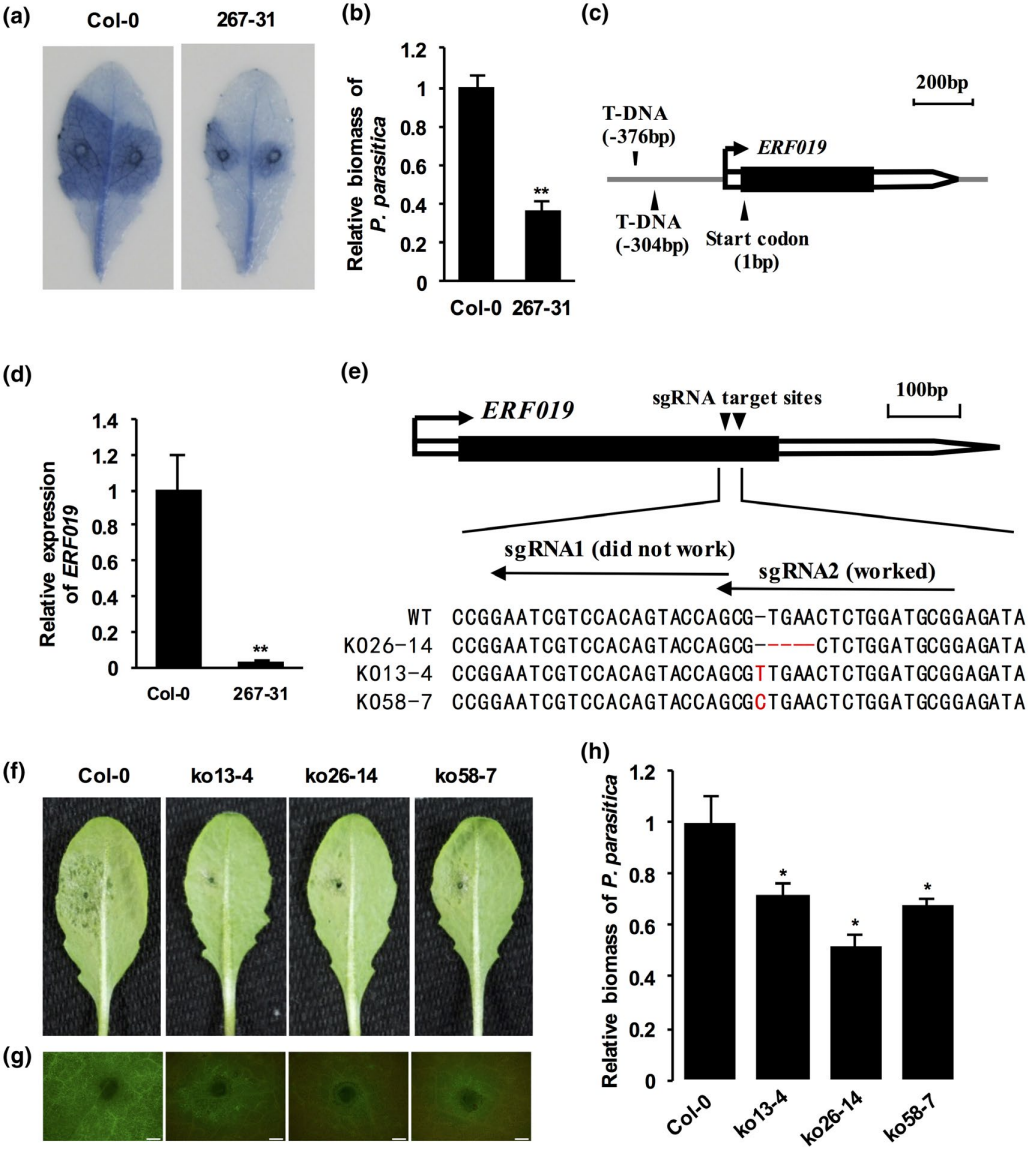
The *erf019* mutants of *Arabidopsis thaliana* limit *Phytophthora parasitica* colonization. (a) Trypan blue staining showing the disease symptoms of the *erf019* T‐DNA insertion mutant 267‐31 and the wild‐type Col‐0 infected with *P. parasitica* strain Pp016. The concentration of zoospore suspensions was adjusted to 200 zoospores/µl. Detached leaves of 4‐week‐old *A. thaliana* seedlings were drop‐inoculated with 10 µl *P. parasitica* zoospores (200 zoospores/µl) and photographed at 3 days postinoculation (dpi). (b) Quantitative reverse transcription PCR (RT‐qPCR) quantification of pathogen colonization. Total genomic DNA from *P. parasitica*‐infected regions was isolated at 2 dpi. Quantitative PCR (qPCR) with primers specific for the *A. thaliana UBC9* gene (*AtUBC*) and the *P. parasitica UBC* gene (*PpUBC*) was used to determine *P. parasitica* biomass in infected plant tissues. The relative *P. parasitica* biomass was calculated by *PpUBC*/*AtUBC* and normalized using the value of Col‐0. (c) Two T‐DNAs were inserted in the promoter region of *ERF019*. (d) RT‐qPCR analysis to quantify the expression of *ERF019* in 2‐week‐old seedlings of *Arabidopsis* mutant 267‐31 and wild‐type Col‐0. *UBC9* was used as the internal control. Bars represent standard errors from three biological replicates and asterisks indicate statistical significance based on *t* test (**p < .01). (e) Targeted indel mutations at the *ERF019* gene. Representative sequences of CRISPR/Cas9‐based knockout mutant alleles identified from transgenic plants expressing sgRNA targeting *ERF019*. (f) Disease symptoms of CRISPR/Cas9‐based knockout mutants and the wild‐type Col‐0 infected with *P. parasitica*. The concentration of zoospore suspensions was adjusted to 200 zoospores/µl. Detached leaves of 4‐week‐old *Arabidopsis* plants were drop‐inoculated with 20 µl *P. parasitica* zoospores (200 zoospores/µl) and photographed at 2 dpi. (g) Pathogen colonization in knockout lines. Detached leaves of 4‐week‐old *Arabidopsis* were drop‐inoculated with 20 µl zoospores of *P. parasitica* transformant 1121, which stably expresses green fluorescent protein (GFP), and visualized under a fluorescence microscope at 2 dpi. Green fluorescence indicates *P. parasitica* hyphae, autofluorescence from leaf tissue is visible as red signal. The white bars indicate 500 µm. (h) Quantification of *P. parasitica* biomass in inoculated leaves of knockout lines by qPCR. Error bars represent *SD*, and asterisks indicate statistical significance based on *t* test (*p < .05; **p < .01). Similar results were obtained from at least three individual experiments

To identify genes that negatively regulate defence against *P. parasitica* infection, we screened nearly 10,000 independent *Arabidopsis* T‐DNA insertion lines (Zhang *et al*., 2005) to identify mutants involved in limiting the colonization of *P. parasitica*. This led to the identification of the mutant 267‐31 (Figure 1a,b). In comparison to wild‐type Col‐0, growth of the pathogen, *P. parasitica* Pp016 (Wang *et al*., 2011b; Zhang *et al*., 2011), was much more restricted in 267‐31 at 3 days postinoculation (dpi) (Figure 1a). Consistent with this finding, quantification of *P. parasitica* colonization in infected *Arabidopsis* leaves revealed less colonization in 267‐31 (Figure 1b).

Thermal asymmetric interlaced (TAIL)‐PCR (Liu *et al*., 1995) was applied to obtain sequences flanking the T‐DNA insertion sites in 267‐31. Sequence analysis showed that there were two T‐DNA insertion sites located 309 and 376 bp upstream of the *ERF019* translation start codon, respectively (Figure 1c). The two T‐DNA insertion fragments were adjacent and in opposite orientations. Quantitative reverse transcription (RT) PCR (RT‐qPCR) analysis showed that the *ERF019* transcript levels were dramatically lower in 267‐31 than in the wild‐type Col‐0 (Figure 1d). Thus, *ERF019* might play a negative role in resistance against *P. parasitica*.

### 2.2 *ERF019* contributes to plant susceptibility to *P. parasitica*

To confirm that *ERF019* contributes to plant susceptibility to *P. parasitica*, we used the CRISPR/Cas9‐mediated genome‐editing tool to knock out the *ERF019* gene. Two target sites in the exon of *ERF019* were chosen (Figure 1e), and the corresponding sgRNA/Cas9 vectors were transformed into the wild‐type Col‐0 via *Agrobacterium tumefaciens*‐mediated transformation. The mutations at the target sites in the CRISPR/Cas9 transformants were examined using PCR and DNA sequencing analysis, which showed that approximately 90% of T_0_ transformants carried mutations at one sgRNA target site while no mutations were found at the other target site. Of three individual homozygous knockout lines chosen for further analysis, two contain a 1‐bp insertion (ko13‐4 and ko58‐7) and one contains a 4‐bp deletion (ko26‐14) in the coding region of *ERF019* (Figure 1e), which results in a frameshift mutation and predicted truncated protein (Figure S1). Phenotypic observations revealed that none of these three *erf019* knockout lines had obvious morphological abnormalities (Figure S2), suggesting *ERF019* is not essential for plant growth and development. When inoculated with 1121, a transformant of Pp016 stably and widely expressing ER‐rendered green fluorescent protein (GFP) under control of the constitutive *Hsp70* promoter of *Bremia lactucae* (Zhang *et al*., 2011), the CRISPR/Cas9‐edited mutants had attenuated *P. parasitica* leaf colonization, with significantly smaller water‐soaked lesions compared with those in the wild‐type Col‐0 (Figure 1f). Microscopic observation showed that fewer GFP‐expressing hyphae colonized the CRISPR/Cas9‐edited mutants compared with the wild‐type Col‐0 (Figure 1g). In addition, quantification of *P. parasitica* biomass revealed that CRISPR/Cas9‐edited mutants exhibited limited colonization of *P. parasitica* (Figure 1h).

**Figure 2:**
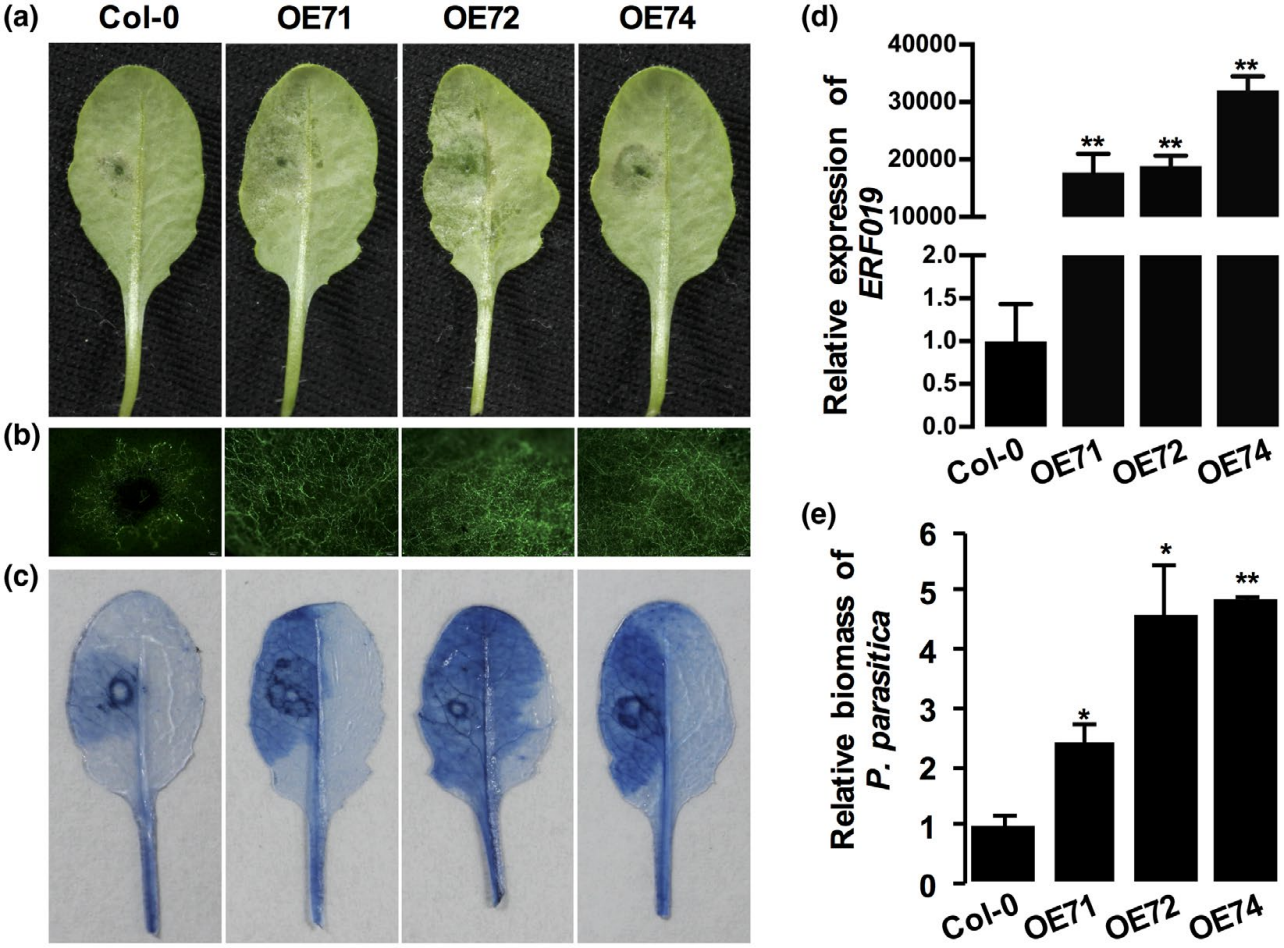
The *ERF019*‐overexpression (*ERF019*‐OE) *Arabidopsis thaliana* plants were more susceptible to *Phytophthora parasitica*. (a) Disease symptoms of *ERF019*‐OE plants infected with 10 µl *P. parasitica* zoospores (200 zoospores/µl) and photographed at 2 days postinoculation (dpi). The concentration of zoospore suspensions was adjusted to 200 zoospores/µl using microscopy. (b) Pathogen colonization on *ERF019*‐OE plants. Detached leaves of 4‐week‐old *A. thaliana* plants were drop‐inoculated with 10 µl zoospores of *P. parasitica* transformant 1121, which stably expresses green fluorescent protein (GFP), and visualized under a fluorescence microscope at 2 dpi. Green fluorescence indicates *Phytophthora* hyphae. (c) Trypan blue staining of *ERF019*‐OE plants infected with *P. parasitica*. Plant cells infected by pathogen were coloured. (D) Quantitative reverse transcription PCR analysis showed the accumulation of *ERF019* transcripts in rosette leaves of *ERF019*‐OE plants. Data represent the ratio of *ERF019* expression between *ERF019*‐OE plants and wild‐type Col‐0. *UBC9* was used as the internal control. Bars represent standard errors from three biological replicates and asterisks indicate statistical significance based on *t* test (**p < .01). (e) Quantification of *P. parasitica* biomass in inoculated leaves of *ERF019*‐OE plants by quantitative PCR. Error bars represent *SD*, and asterisks indicate statistical significance based on *t* test (*p < .05; **p < .01). Similar results were obtained from at least three individual experiments

To further clarify the function of *ERF019* in plant susceptibility, we transformed an *ERF019*‐overexpression (OE) construct, in which *ERF019* expression is under the control of constitutive cauliflower mosaic virus (CaMV) 35S promoter, into the Col‐0 background. Three *ERF019*‐OE lines, OE71, OE72, and OE74, were selected for further analysis following confirmation by RT‐qPCR analysis that the *ERF019* transcript levels were significantly increased in these lines (Figure 2d). Infection assays with *P. parasitica* revealed that *ERF019*‐OE plants were more susceptible than the wild‐type Col‐0. Two days after pathogen infection, *ERF019*‐OE lines developed much larger water‐soaked lesions than the wild‐type Col‐0 (Figure 2a). Heavier hyphal colonization was also visible in *ERF019*‐OE plants when infected with the *P. parasitica* transformant 1121, which stably expresses GFP (Figure 2b). In addition, both a trypan blue staining assay and a quantification of *P. parasitica* biomass revealed that *ERF019*‐OE plants exhibited enhanced disease susceptibility to *P. parasitica* (Figure 2c,e). These results confirmed that *ERF019* negatively regulates plant resistance to *P. parasitica*.

### 2.3 *ERF019* expression is induced on *P. parasitica* infection

**Figure 3:**
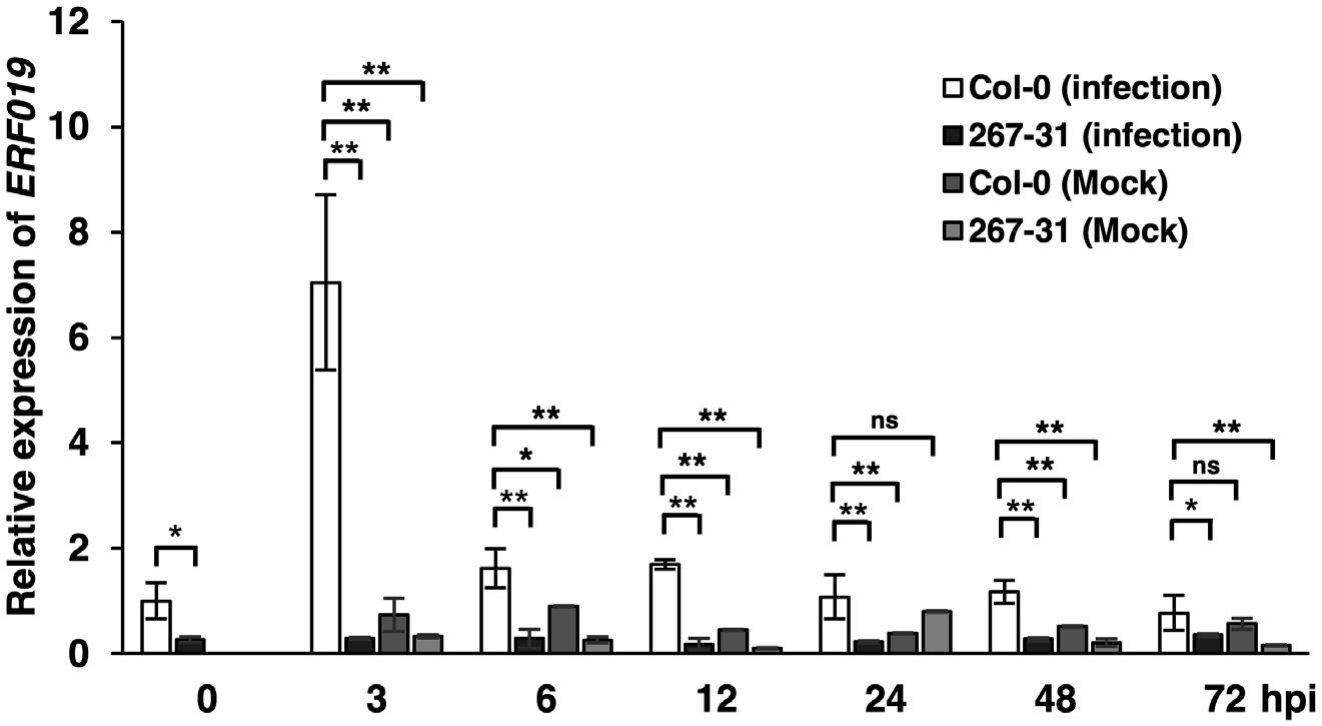
Quantitative reverse transcription PCR analysis for the expression of *ERF019* in *Arabidopsis thaliana* Col‐0 and the T‐DNA insertion mutant 267‐31 at different time points after *Phytophthora parasitica* inoculation. *AtUBC9* was used as the internal control. Bars represent standard errors from three biological replicates and asterisks indicate statistical significance based on *t* test (*p < .05; **p < .01; ns, not significant). 0 h, uninfected leaves; hpi, hours postinoculation

To examine whether the expression of *ERF019* is responsive to *P. parasitica* infection, we used RT‐qPCR to measure the *ERF019* expression levels in *P. parasitica*‐inoculated leaves at different time points. The results showed that in Col‐0, *ERF019* expression was highly induced at 3 hours postinoculation (hpi) and slightly induced at 6 and 12 hpi compared with that in the uninfected leaves and the mock‐inoculated controls. The expression levels of *ERF019* at 24 hpi were decreased to a level similar to that of the uninfected leaves, though it appeared a little higher than that in the mock‐inoculated controls. These observations indicate that *ERF019* is responsive to *P. parasitica* infection. In contrast, the expression level of *ERF019* in the 267‐31 mutant was significantly lower than that in the wild type, both in the uninfected and infected leaves, though the mutant still produced few detectable transcripts (Figure 3).

### 2.4 Nuclear localization of ERF019 is required for its susceptibility function

**Figure 4:**
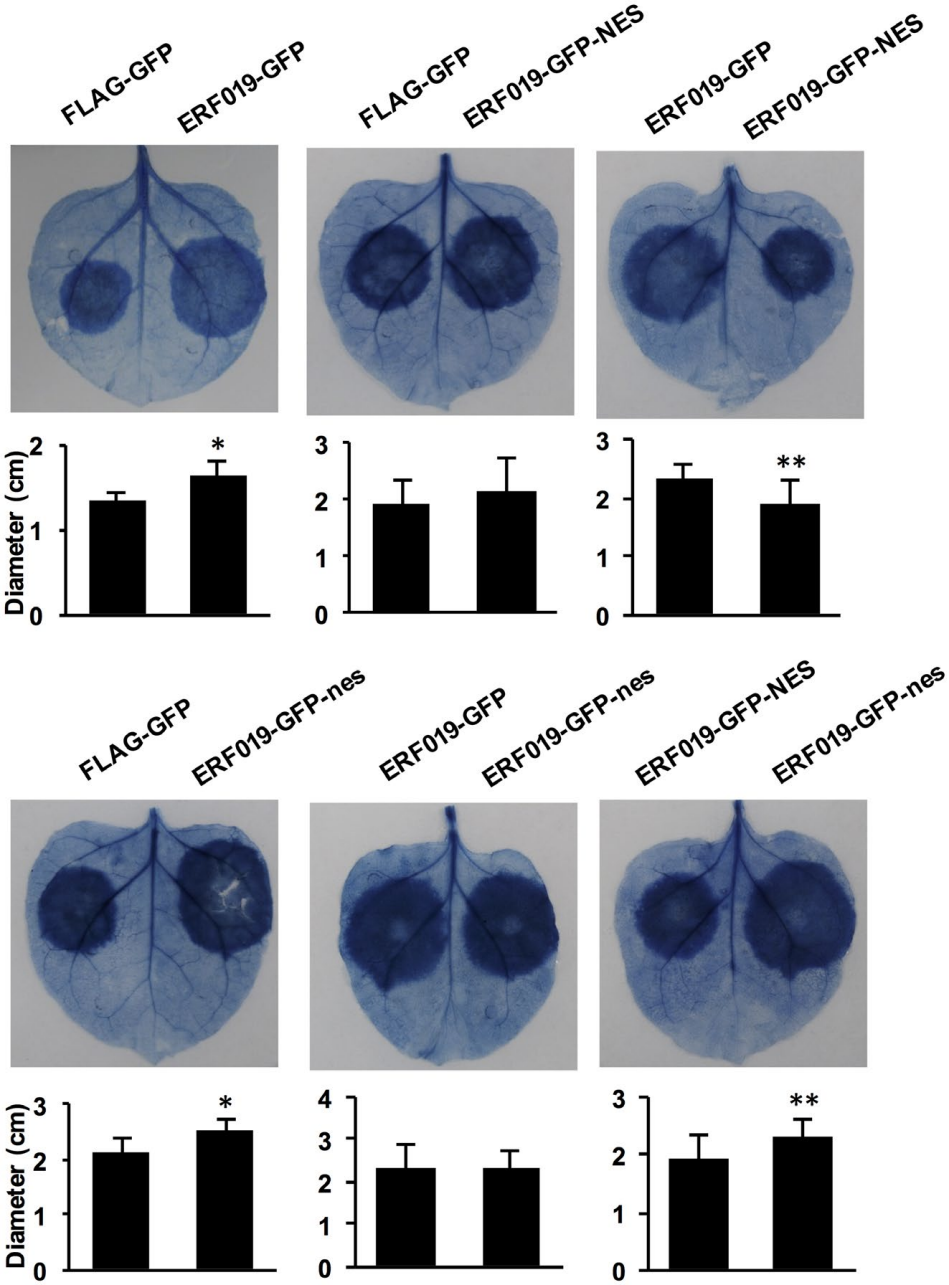
Nuclear localization is required for ERF019 to promote *Phytophthora parasitica* growth in *Nicotiana benthamiana. P. parasitica* colonization of *N. benthamiana* leaves expressing FLAG‐GFP, ERF019‐GFP, ERF019‐GFP‐NES or ERF019‐GFP‐nes. *Agrobacterium tumefaciens* GV3101 cells carrying FLAG‐GFP, ERF019‐GFP, ERF019‐GFP‐NES or ERF019‐GFP‐nes were infiltrated into leaves of *N. benthamiana*, and infiltrated leaves were challenged with 10 µl *P. parasitica* Pp016 zoospores (200 zoospores/µl) 48 hr after infiltration. The concentration of zoospore suspension was adjusted to 200 zoospores/µl. Disease symptoms were observed 2 days postinoculation and leaves were stained with trypan blue. At least 10 leaves were used for the test. Error bars represent *SD*, and asterisks indicate statistical significance based on a two‐tailed *t* test (*p < .05; **p < .01). Similar results were obtained for at least three individual experiments

ERF019 contains an APETALA2 (AP2) domain and is, therefore, predicted as a member of the ethylene‐responsive factor (ERF)/AP2 transcription factor family. We transiently co‐expressed the ERF019‐GFP fusion construct with the nucleus marker H2B‐mCherry in *Nicotiana benthamiana* leaves and found that ERF019‐GFP can be observed in both nucleus and cytoplasm (Figure S3). To confirm that the nuclear localization of ERF019 is necessary for its function in promoting pathogen infection, we altered the subcellular localization of ERF019 by fusing it with a nuclear export sequence (NES). A construct expressing ERF019 fused with a mutant NES (nes) was used as a control. We first transiently expressed the ERF019‐GFP‐NES and ERF019‐GFP‐nes chimeric proteins under the control of the CaMV 35S promoter in *N. benthamiana* leaves and observed the subcellular localization by fluorescence microscopy. ERF019‐GFP‐NES was clearly exported to the cytosol and was barely detectable in the nucleus, while ERF019‐GFP‐nes displayed a localization pattern similar to that of ERF019‐GFP (Figure S4), which indicates that the NES was functional. Next, to examine whether the nuclear export of ERF019 affects its role in plant susceptibility, we transiently expressed these fusion proteins in *N. benthamiana* leaves and challenged the leaves with *P. parasitica*. Notably, leaves transiently expressing ERF019‐GFP displayed significantly larger infection lesions compared with those expressing the FLAG‐GFP control (Figure 4), which is consistent with the role of ERF019 as a negative regulator of plant immunity. ERF019‐GFP‐NES, but not ERF019‐GFP‐nes, lost its ability to enhance *P. parasitica* colonization of *N. benthamiana* (Figure 4). These results suggest that the nuclear localization of ERF019 is required for its function in promoting colonization of *P. parasitica*.

### 2.5 Expression of defence marker genes is up‐regulated in the *erf019* mutant

**Figure 5:**
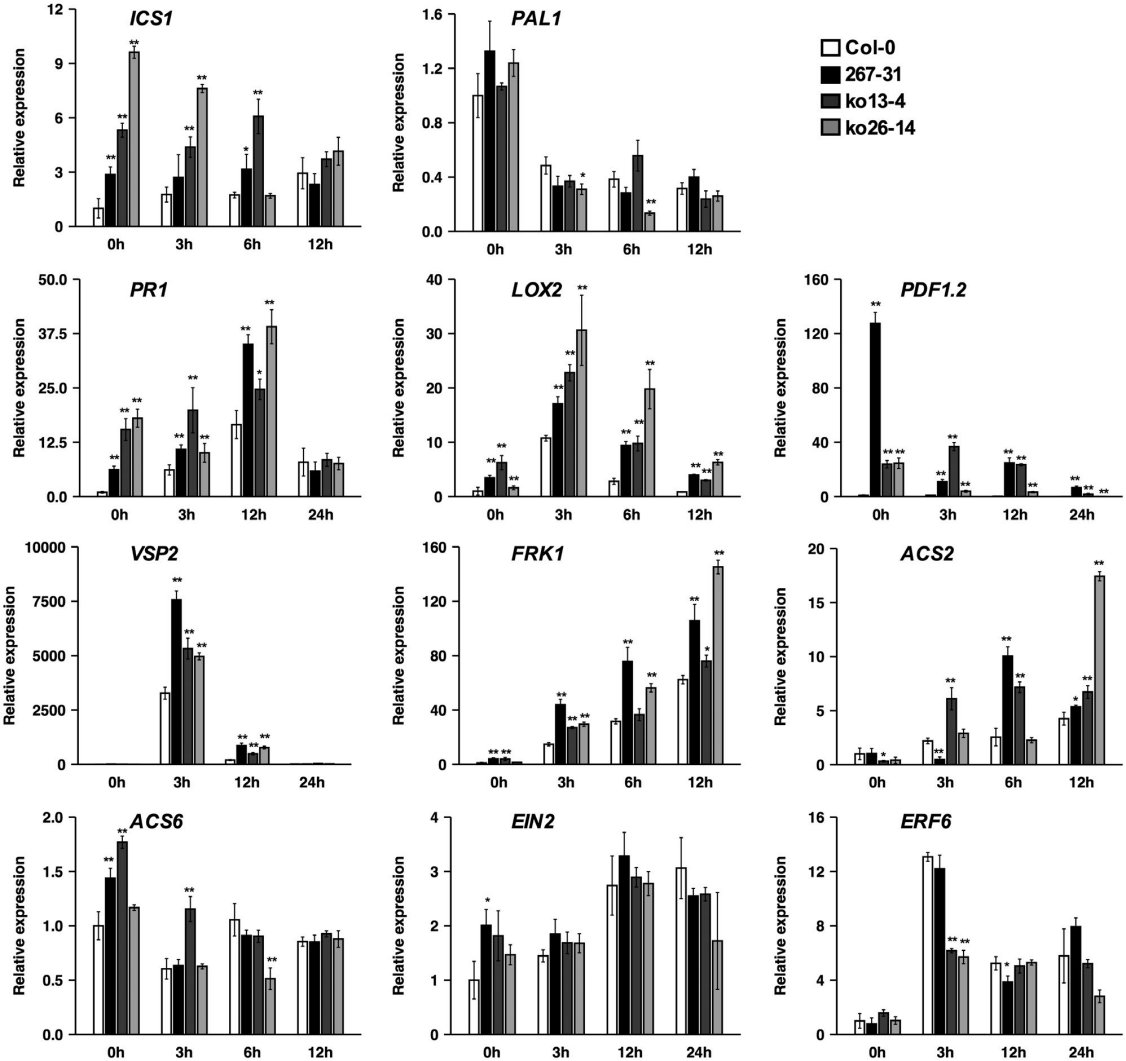
Defence marker gene expression in the *erf019* mutants of *Arabidopsis thaliana*. Transcript levels of defence‐related marker genes in the *erf019* mutants (267‐31, ko13‐4, and ko26‐14) and Col‐0 were evaluated by quantitative reverse transcription PCR (RT‐qPCR) at different times postinoculation. RT‐qPCR data are presented as relative transcript level for genes: *ICS1* and *PAL1*, two marker genes for salicylic acid (SA) biosynthesis; *PR1*, a marker for the SA signalling pathway; *LOX2*, a marker for jasmonic acid (JA) biosynthesis; *PDF1.2* and *VSP2*, involved in the JA signalling pathway; *FRK1*, a marker gene for the PAMP‐triggered immunity (PTI) pathway; *ACS2* and *ACS6*, two marker genes for ethylene (ET) biosynthesis; and *ERF6* and *EIN2*, involved in the ET signalling pathway. *AtUBC9* was used as the internal control and transcript levels relative to Col‐0 plants are displayed. Bars represent *SE* from three biological replicates and asterisks indicate statistical significance based on a two‐tailed *t* test (*p < .05; **p < .01). 0 h, uninfected leaves

Because *ERF019* negatively regulates plant resistance to *P. parasitica*, we examined the potential role of *ERF019* in known defence pathways by measuring the expression of marker genes associated with these pathways in 267‐31 and the CRISPR/Cas9‐edited *erf019* mutant lines (ko13‐4, ko26‐14). *ICS1* (*Isochorismate synthase* *1*) and *PAL1* (*Phenylalanine ammonia lyase 1*) are involved in the synthesis of SA (Wildermuth *et al*., 2001), and *PR1* (*Pathogenesis‐related gene 1*) is a well‐established marker gene for the SA signalling pathway (Uknes *et al*., 1992). *LOX2* (*Lipoxygenase* *2*) is involved in the synthesis of jasmonic acid (JA) (Sasaki *et al*., 2001), and *PDF1.2* (*Plant defensin gene 1.2*) and *VSP2* (*Vegetative storage protein 2*) are genes that respond to JA (Pieterse *et al*., 2009). *FRK1* (*Flg22‐induced receptor‐like kinase 1*) is a core gene that is induced by a conserved 22 amino acid epitope from bacterial flagellin (flg22), and is frequently used to monitor PTI (Shan *et al*., 2008). *ACS2* and *ACS6*, two members of the *ACS* gene family that encode 1‐amino‐cyclopropane‐1‐carboxylase synthase, are involved in the ethylene biosynthesis (Van der Straeten *et al*., 1992), and *EIN2* (*Ethylene‐insensitive protein 2*) is an important regulator in the ET signalling pathway (Alonso *et al*., 1999) and *ERF6* (*Ethylene‐responsive factor 6*) is an ET‐related signalling gene (Moffat *et al*., 2012). An RT‐qPCR assay showed that the expression of defence‐related genes was altered in the *erf019* mutants compared with the wild‐type Col‐0. In *P. parasitica*‐inoculated plants, the expression levels of *ICS1*, *PR1*, *VSP2*, *LOX2, PDF1.2*, and *FRK1* in 267‐31 and the CRISPR/Cas9‐edited lines were significantly higher than that in the wild‐type Col‐0 (Figure 5). The expression of *PAL1* was down‐regulated on infection, and showed similar level between mutants and the wild‐type Col‐0 (Figure 5). However, for the marker genes in the ET signalling pathway, the expression levels of *ACS6*, *EIN2*, and *ERF6* appeared similar between mutants and the wild type, although *ACS2* was induced to higher levels in mutants at some time points (Figure 5). Taken together, these results indicate that *ERF019* may play an important role in the SA and JA defence signalling pathways but not the ET signalling pathway.

### 2.6 *ERF019* suppresses PTI responses

**Figure 6:**
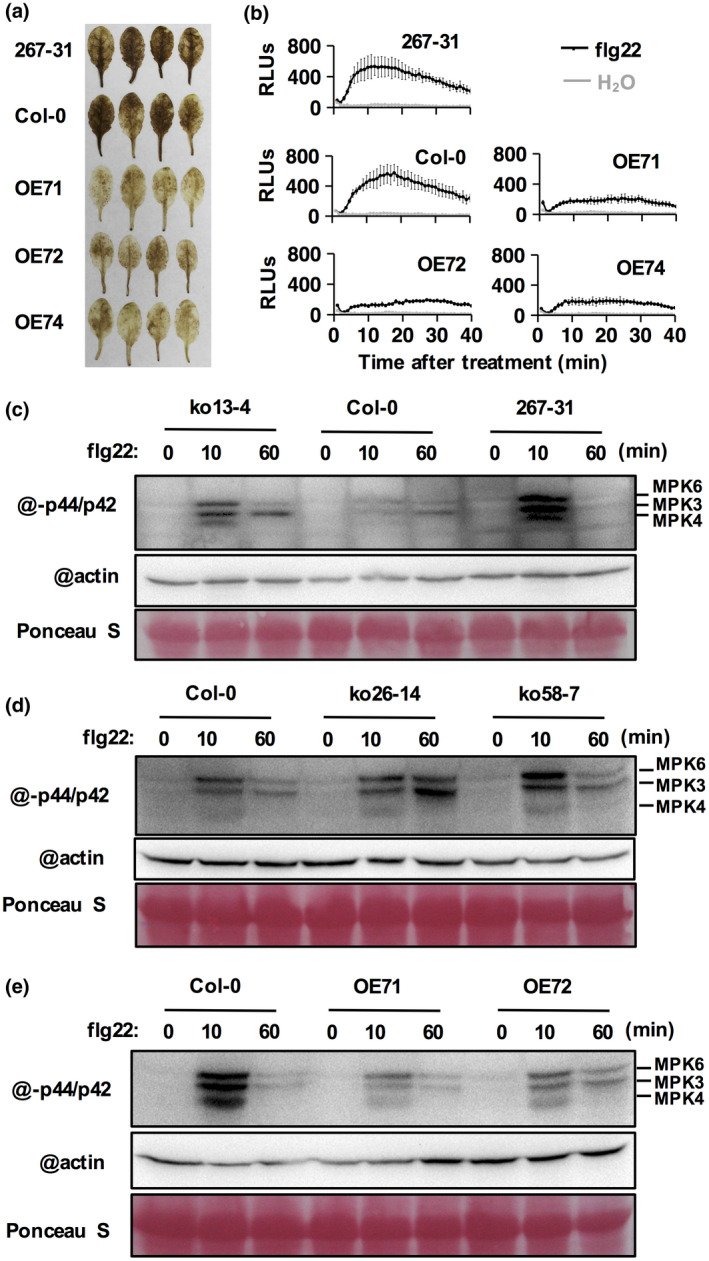
Flg22‐triggered immunity in *erf019* mutants and *ERF019* overexpression lines. (a) Detection of hydrogen peroxide by 3,3′‐diaminobenzidine‐ tetrahydrochloride (DAB) staining. Leaves from the *erf019* mutant (267‐31) and *ERF019* overexpression lines (OE71, OE72, and OE74) were infiltrated with 1 μM flg22; hydrogen peroxide was detected 24 hr later. (b) The reactive oxygen species (ROS) burst in the 267‐31 mutant and *ERF019* overexpression lines after treatment with 1 μM flg22. Total relative luminescent units (RLUs) were detected over 30 min using leaf discs of 4‐week‐old plants. (c)–(e) Immunoblotting of phosphorylated MAP kinase on flg22 treatment in *erf019* mutants (ko13‐4, ko26‐14, ko58‐7, and 267‐31), *ERF019*‐OE lines (OE71 and OE7*2*), and wild‐type Col‐0. Samples were collected at 0, 10, and 60 min after flg22 treatment, total protein was extracted and analysed by immunoblots using antibodies against phospho‐p44/42 MAPK and actin. Similar results were obtained in two independent experiments

Perception of flg22 triggers a series of immunity responses, including an oxidative burst, rapid and transient accumulation of ROS, and the activation of MAP kinases. *ERF019* was reported to be highly induced by flg22 (Sano *et al*., 2014; Huang *et al*., 2019), which suggests that *ERF019* may be involved in flg22‐triggered immunity. To test this hypothesis, we used 3,3′‐diaminobenzidine‐ tetrahydrochloride (DAB) staining to detect hydrogen peroxide accumulation in Col‐0, the *erf019* mutant, and *ERF019*‐OE lines on flg22 treatment. Although no obvious difference was observed between the *erf019* mutant and Col‐0, our results revealed that the flg22‐induced accumulation of hydrogen peroxide was impaired in *ERF019*‐OE lines (OE71, OE72, and OE74) (Figure 6a). We also monitored the flg22‐induced accumulation of ROS in *erf019* mutants and *ERF019*‐OE lines. The results of this analysis were similar to those of DAB staining: ROS accumulation was impaired in *ERF019*‐OE lines but not in the *erf019* mutants (Figure 6a). Moreover, we also found that the activation of MAPK3, MAPK4, and MAPK6, on flg22 treatment, was much stronger in both 267‐31 and CRISPR/Cas9 mutants (ko13‐14, ko26‐14, and ko58‐7) than in Col‐0 (Figure 6c,d), and the PAMP‐triggered MAPK activation was compromised in *ERF019*‐OE lines (OE71 and OE72) (Figure 6e). These results suggest that *ERF019* negatively regulates PAMP‐triggered immunity.

**Figure 7:**
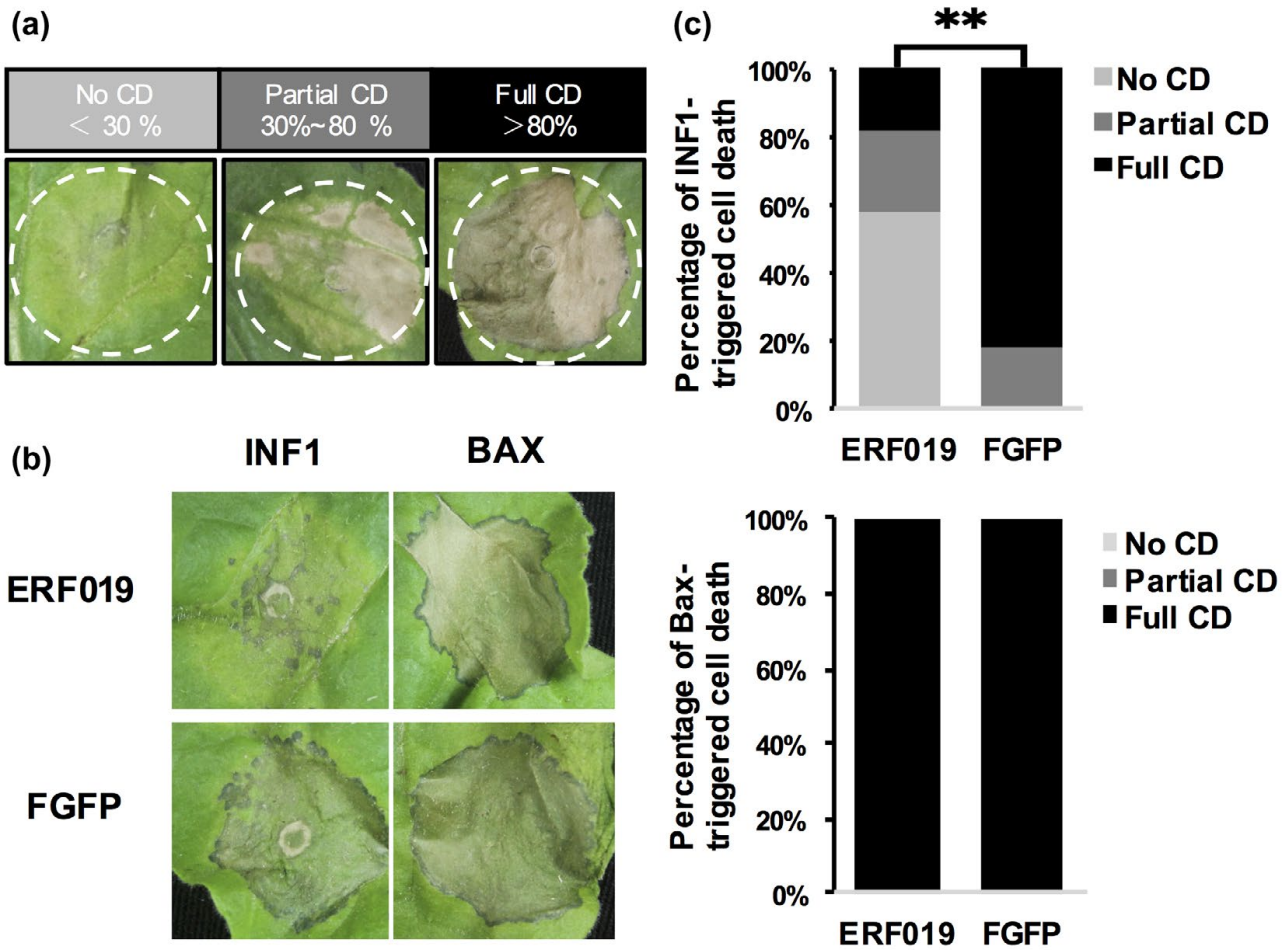
*ERF019* suppresses INF1‐triggered cell death in *Nicotiana benthamiana*. (a) The degree of elicitor‐induced programmed cell death was categorized into three classes: no cell death (cell death area accounts for less than 30% of the injected area), partial cell death (cell death area accounts for 30%–80% of the injected area), and full cell death (cell death area accounts for more than 80% of the injected area). (b) Expression of *ERF019* suppressed necrosis triggered by *Phytophthora infestans* PAMP elicitor INF1 but not Bax. *Agrobacterium tumefaciens* GV3101 cells carrying 35S::FLAG‐GFP or 35S::ERF019 were mixed with cells carrying elicitor constructs and were co‐infiltrated into 5‐week‐old *N. benthamiana* leaves. Phenotypic changes were monitored at 4 days postinfiltration. (c) Effect of *ERF019* expression on necrosis triggered by INF1 and Bax. At least 30 infiltration sites were examined. Asterisks indicate statistical significance based on a two‐tailed *t* test (**p < .01). Similar results were obtained in at least two independent experiments

To further demonstrate whether *ERF019* negatively regulates PTI triggered by *Phytophthora* elicitors, we tested whether it could inhibit INF1‐induced necrosis. We first transiently overexpressed the ERF019 protein in *N. benthamiana* leaves using *A. tumefaciens*‐mediated transformation and found that *ERF019* did not induce necrosis after monitoring for up to 7 days postinfiltration (Figure S5). Next, we co‐infiltrated mixtures of *A. tumefaciens* cultures carrying constructs of elicitors and either ERF019 or FLAG‐GFP into 5‐week‐old *N. benthamiana* leaves. An HR in *N. benthamiana* leaves was observed 4 days postinfiltration, and the responses were classified into three categories according to the degree of response: no cell death, partial cell death, and full cell death (Figure 7a). Interestingly, *ERF019* expression significantly suppressed the HR induced by INF1 compared with the control FLAG‐GFP (Figure 7b,c). We also co‐infiltrated *ERF019* with the proapoptotic protein elicitor Bax and found that *ERF019* did not significantly inhibit Bax‐induced cell death (Figure 7b,c). These results suggest that *ERF019* specifically suppresses *Phytophthora* elicitor INF1‐triggered cell death in *N. benthamiana*. Taken together, we demonstrated that *ERF019* negatively regulates plant resistance by inhibiting PTI.

## 3 DISCUSSION

The plant diseases caused by *Phytophthora* spp. pose a great threat to agriculture, highlighting the importance of studies on the mechanisms of plant resistance. Previous studies based on forward genetics or map‐based cloning technology have characterized dozens of R genes, which are commonly used in crop resistance breeding. However, R gene‐mediated resistance, also described as effector recognition‐based resistance, has been frequently overcome by new pathogen races. In contrast, disabling plant disease susceptibility genes (negative regulators of plant resistance) may provide a novel way to achieve durable and broad‐spectrum resistance. For example, loss‐of‐function of the *MLO* gene confers broad‐spectrum resistance to the powdery mildew fungus (Jorgensen, 1992; Büschges *et al*., 1997; Piffanelli *et al*., 2002). The *mlo* allele has been introduced into European spring barley cultivars, and this has provided robust resistance for nearly four decades (Jorgensen, 1992; Lyngkjær and Carver, 2000), suggesting the great potential of disabling negative regulators in improving crop disease resistance.

Here, we showed the successful use of the compatible system between *P. parasitica* and *A. thaliana* to identify negative regulators. We identified the T‐DNA insertion mutant 267‐31, which was demonstrated to be resistant to *P. parasitica* without obvious inhibition of growth (Figure 1). Multiple *erf019* frameshift mutants generated by CRISPR/Cas9 technology consistently showed enhanced resistance to *P. parasitica*, while *ERF019* overexpression lines were more susceptible (Figures 1 and 2). These results indicate that *ERF019* negatively regulates plant resistance to *P. parasitica*.

The ERF family is a large family of transcription factors in plants, with up to 122 members in *Arabidopsis* and 139 members in rice (Nakano *et al*., 2006). ERFs are involved in diverse developmental processes and various responses to environmental stimuli, such as pathogen attack, drought, salt, wounding, UV irradiation, and extreme temperature (Tsutsui *et al*., 2009; Liu *et al*., 2012; Licausi *et al*., 2013; Maruyama *et al*., 2013). *ERF019* was classified into phylogenetic Group II of the *Arabidopsis* ERF family (Nakano *et al*., 2006). There are 15 members in this group, which are further classified into three subgroups: IIa, IIb, and IIc. Most genes in subgroups IIa and IIb have been shown to play crucial roles in biotic and abiotic stress responses. For example, overexpression of *DEAR1*, a subgroup IIa gene, rendered *Arabidopsis* more resistant to *Pseudomonas syringae* infection and less tolerant to freezing (Tsutsui *et al*., 2009). Furthermore, all six genes of subgroup IIa induce cell death in tobacco (Ogata *et al*., 2013). *ERF15*, a subgroup IIb gene, has been reported to be a negative regulator of salt and drought tolerance (Lee *et al*., 2015). Here, we found that the subgroup IIc gene *ERF019* negatively regulates plant resistance to *P. parasitica*. It has also been reported that overexpression of *ERF019* delays plant growth and senescence, enhances drought resistance, and increases plant susceptibility to *Botrytis cinerea* and *P. syringae* (Scarpeci *et al*., 2017; Huang *et al*., 2019). These observations suggest that ERF family subgroup II members may play important roles in plant resistance and abiotic stress, and functional analysis of their orthologous genes in crops will provide potential gene resources for breeding for disease resistance.

Plant PTI responses, including ROS burst, callose deposition, MAP kinase activity, and defence gene induction, are critical for plants to repel pathogen attacks (Bigeard *et al*., 2015). We show here that the induction of the PTI‐related marker gene *FRK1* in the 267‐31 mutant on *P. parasitica* infection was stronger than that in the wild type Col‐0 (Figure 5). Meanwhile, flg22‐induced activation of the MPK3, MPK4, and MPK6 was enhanced in both the 267‐31 mutant and *erf019* knockout lines when compared with Col‐0, and was compromised in *ERF019*‐OE lines (Figure 6). The flg22‐induced ROS burst and hydrogen peroxide accumulation were also significantly suppressed in the leaves of *ERF019*‐OE plants (Figure 6), which is consistent with the previous report that flg22‐induced callose deposition was significantly impaired in *ERF019*‐OE plants (Huang *et al*., 2019). Furthermore, we showed that transient overexpression of *ERF019* in *N. benthamiana* suppresses *Phytophthora* PAMP elicitor *INF1*‐activated cell death (Figure 7). These results demonstrate the critical role of *ERF019* in negatively regulating PTI responses.

Plant cell death plays a central role in interactions with hemibiotrophic pathogens, such as *Phytophthora* species, considering that these pathogens initially develop haustoria to acquire nutrients from living host cells and then subsequently switch to a necrotrophic lifestyle, resulting in the death of the host plant (Lamour *et al*., 2012). INF1‐like proteins are a family of secreted elicitins, which exist widely in *Phytophthora*. It is reasonable to hypothesize that *ERF019* may negatively regulate plant resistance by suppressing cell death, which facilitates the growth of *P. parasitica* during plant infection. Meanwhile, *ERF019* cannot inhibit Bax‐induced cell death, like *P. sojae* effector Avh238 that was reported to inhibit INF1‐ but not Bax‐induced cell death (Wang *et al*., 2011a). Bax is a proapoptotic member and can translocate into the mitochondrial membrane and trigger the apoptotic process, some features of which resemble plant programmed cell death (Ihara‐Ohori *et al*., 2007). However, compared to Bax‐induced cell death, the recognition of INF‐1 and the downstream pathway of INF1‐triggered cell death may possess some unique features, some of which may be regulated by *ERF019*. Because the overexpression of *ERF019* leads to attenuation of PTI responses, and the silencing of *N. benthamiana* receptor‐like kinase gene *SERK3*, which encodes a homolog of *Arabidopsis* BAK1 and plays a key role in PTI by suppressing INF1‐induced cell death (Chaparro‐Garcia *et al*., 2011), it is likely that *ERF019* suppresses INF1‐activated cell death by interfering with the PTI signalling pathway.

Interestingly, the negative regulator *ERF019* was induced during *P. parasitica* infection. Moreover, *ERF019* was also highly induced by flg22 (Huang *et al*., 2019). These observations suggested that *ERF019* can be induced during PTI, which in turn inhibits PTI. Previous researchers showed that plant recognition of PAMPs induces both positive and negative PTI signalling pathways. For example, the PAMP‐induced MEKK1, MEKK1/2, and MPK4 signalling cascades negatively mediate plant defence responses (Ichimura *et al*., 2006; Mészáros *et al*., 2006; Suarez‐Rodriguez *et al*., 2007; Gao *et al*., 2008; Qiu *et al*., 2008; Pitzschke *et al*., 2009). Thus, *ERF019* may be involved in a negative feedback loop that balances growth and resistance.

Our results also showed that the SA biosynthesis‐related gene *ICS1*, SA signalling pathway marker gene *PR1*, JA signalling marker gene *VSP2* and *PDF1.2* as well as JA biosynthesis‐related gene *LOX2* were up‐regulated in the *erf019* mutant, indicating that the SA and JA signalling pathways are coupled through *ERF019*. It has been reported that SA is responsible for plant defence against biotrophs, whereas JA or ET is responsible for defence against necrotrophs (Bostock, 2005). However, both the SA and JA signalling pathways have been shown to contribute to basal resistance against *P. parasitica* (Attard *et al*., 2010). Interference with SA, JA, or ET signalling in the *eds1*, *eds5*, *pad4*, *sid2*, *ein2*, *etr1*, and *jar1* mutants and *NahG* transgenic plants enhanced plant susceptibility to *P. parasitica* (Attard *et al*., 2010). Moreover, the *Arabidopsis thaliana Resistant to Phytophthora 5* gene (*AtRTP5*), which encodes a WD40 repeat domain‐containing protein, has been reported to negatively regulate plant resistance to *P. parasitica* by interfering with the JA and SA signalling pathways (Li *et al*., 2020). In addition, *ERF019* was shown to be induced by OPDA, a cyclopentenone precursor of JA (Taki *et al*., 2005). The function of ERF019 may be repressed by NINJA (the transcriptional co‐repressor Novel INteractor of JAZ), a negative regulator of JA signalling, through protein–protein interaction (Huang *et al*., 2019). These results suggest a potential role of ERF019 in the JA signalling pathway, which is subjected to complex positive and negative regulation and is coupled with the SA signalling pathway.

Loss of function of a negative regulator of plant resistance may constitutively activate defence responses and reduce plant fitness (Tian *et al*., 2003; Denancé *et al*., 2013; Huot *et al*., 2014). For example, loss of function of the *MPK4* gene results in a dwarf phenotype, which is accompanied by elevated SA levels and constitutive expression of pathogenesis‐related genes (Petersen *et al*., 2000). The *mekk1* mutant and *mkk1 mkk2* double mutant also display constitutive defence responses and reduced plant growth (Gao *et al*., 2008). However, *erf019* plants exhibit resistance to *P. parasitica* without altered plant growth. Consistent with this phenotype, RT‐qPCR results showed that the expression of *ERF019* was low in adult rosette leaves under normal conditions (Figure 3), suggesting that *ERF019* may not be necessary for plant growth. Furthermore, the expression of defence‐related marker genes was just slightly up‐regulated in the *erf019* mutant in the absence of pathogen infection (Figure 5). In addition, after flg22 treatment, activation of MAP kinase was stronger in the *erf019* mutants than in the wild‐type Col‐0 and attenuated in the *ERF019*‐OE plants when compared to the wild‐type Col‐0 after 10 min of treatment (Figure 6c,d,e). These observations show that PTI responses seem to be amplified in the *erf019* mutant without strong constitutive induction of the expression of pathogenesis‐related genes, thus increasing resistance without influencing growth.

ERF019 contains a conserved AP2/ERF DNA‐binding domain at the N‐terminus. There was no conserved motif identified at the C‐terminus of ERF019 based on multiple sequence alignment analyses of ERF family members (Nakano *et al*., 2006). The potentially accumulated proteins in the CRISPR/Cas9‐edited *erf019* mutant lines, which are predicted to be truncated due to the frameshift mutations from the codon for the 109th amino acid, lost ability to negatively regulate resistance, indicating a key role of the C‐terminus of ERF019 in immune function. The regions outside the DNA‐binding domain in ERF proteins are generally involved in protein modification and protein–protein interactions, and are important for their nuclear localization and transcriptional activities (Nakano *et al*., 2006; Licausi *et al*., 2013). For example, the C‐terminal activation domain, but not the N‐terminal DNA binding domain of ERF protein TINY, interacts with and antagonizes BRASSINOSTERIOID INSENSITIVE1‐ETHYL METHANESULFONATE SUPRESSOR1 (BES1) in the regulation of drought response (Xie *et al*., 2019). The transcriptional repressor Novel INteractor of JAZ (NINJA) interacts with ERF019 and represses its function (Huang *et al*., 2019). We speculate that the C‐terminus of ERF019 mediates interaction by other unknown protein factors to regulate plant immunity. Future efforts to identify the ERF019‐interacting proteins will be useful in understanding the underlying mechanisms of ERF019 in regulating plant immunity.

**Figure 8:**
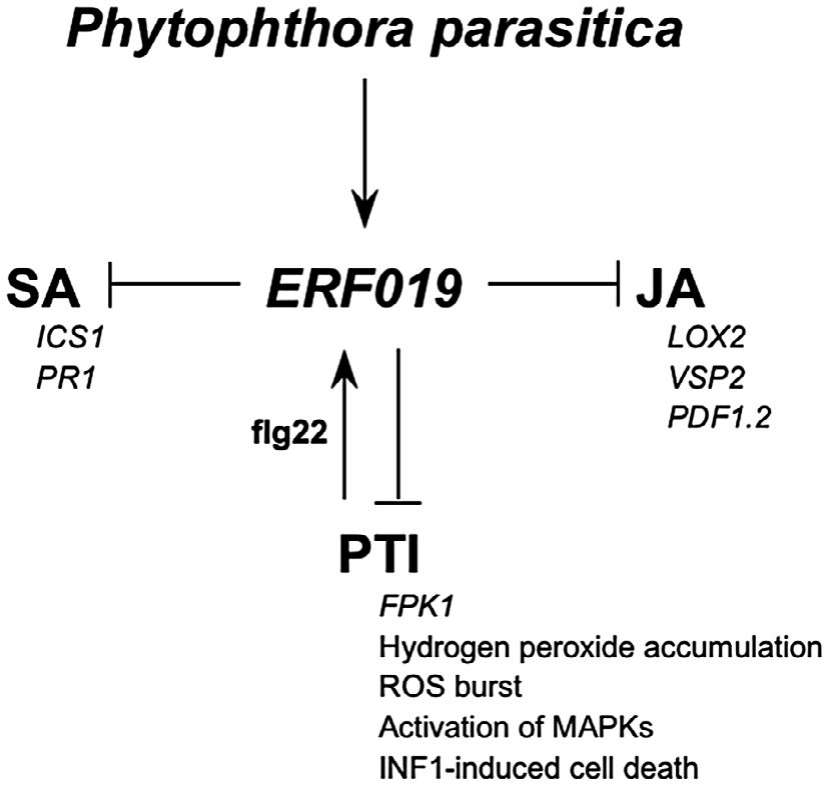
Proposed model for the role of *ERF019* in plant immunity. The PAMP‐triggered immunity (PTI) and multiple defence pathways, including the salicylic acid (SA) and jasmonic acid (JA) signal pathways, are induced on infection or flg22‐treatment. *ERF019* is also induced on infection by *Phytophthora parasitica* but suppresses PTI as well as SA and JA signal pathways, suggesting it plays an important role in the negative feedback loop that balances growth and resistance

Based on our study, we propose that *ERF019* plays an important role in the negative feedback loop that balances growth and resistance on pathogen infection by suppressing PTI and SA/JA defence responses (Figure 8). Further identification of target genes regulated by *ERF019* will provide insights into the mechanisms of the negative feedback loop. Identification of loss‐of‐function alleles of *ERF019* and its homologs in crops is a potential strategy for breeding crops with durable resistance.

## 4 EXPERIMENTAL PROCEDUREES

### 4.1 Plant materials and growth conditions

The *Arabidopsis* T‐DNA insertion lines were generated (Zhang *et al*., 2005) and kindly provided by Dr Jianru Zuo. *Arabidopsis* ecotype Col‐0 and T‐DNA insertion mutants used in this study were grown at 23°C with 14 hr of light per 24 hr. *N. benthamiana* plants were also grown at 23°C with 14 hr of light per 24 hr.

### 4.2 Pathogen growth and infection assays

*P. parasitica* strain Pp016 was originally isolated from diseased tobacco plants in Queensland, Australia (Wang *et al*., 2011b; Zhang *et al*., 2011), and 1121 is a transformant of *P. parasitica* Pp016 stably expressing ER‐rendered GFP under the control of the constitutive *Hsp70* promoter of *B. lactucae*. The transformant 1121 remained pathogenic on *A. thaliana* and tobacco plants, similar to the wild‐type strain Pp016 (Zhang *et al*., 2011). The GFP in *P. parasitica* 1121 is constitutively and widely expressed in the cytoplasm, allowing easy monitoring during colonization of host plants. *P. parasitica* culture conditions, zoospore production, and the detached leaf inoculation assays were performed as described (Wang *et al*., 2011b). For the pathogenicity assay, the detached fully expanded apical leaves from approximately 4‐week‐old *A. thaliana* plants were wounded with a toothpick and the zoospore suspensions were adjusted to a concentration of 200 zoospores/µl using microscopy and applied as droplets at the wounding sites to ensure infection. To observe the lesion size more clearly, trypan blue was used to stain the death plant cells in lesions of inoculated leaves as described (Li *et al*., 2019). *A. thaliana* leaves infected with the *P. parasitica* transformant 1121 were observed with the OLYMPUS BX51 fluorescence microscope (with excitation at 450–480 nm and emission at 515 nm) to detect *P. parasitica* hyphae (green fluorescence) at 2 dpi. For pathogen biomass, three biological replicates were performed with at least eight leaves per replicate. Primers used for pathogen biomass were listed in Table S1. Disease severity was evaluated based on the lesion sizes on detached leaves and the extent of pathogen colonization.

### 4.3 TAIL‐PCR and RT‐qPCR assays

TAIL‐PCR was performed as described (Liu *et al*., 1995). For RT‐qPCR assays, three biological replicates were used. Total RNA from the whole leaves before and after *P. parasitica* inoculation was extracted using TRIzol reagent (Invitrogen). One microgram of total RNA was used to perform reverse transcription using the PrimeScript RT Reagent Kit with gDNA Eraser (TAKARA). For real‐time qPCR analysis, 0.5 µl of the first‐strand cDNA reaction products was used as template in a reaction with Ultra SYBR Mixture (CWBIO) under the following conditions: 95°C for 10 min, and 40 cycles of 95°C for 15 s and 60°C for 30 s. The fold changes in target gene expression were normalized using *UBC9* as the internal control. Primers used for RT‐qPCR are listed in Table S1.

### 4.4 CRISPR/Cas9‐based knockouts and overexpression of *ERF019* in *Arabidopsis*

For the 35S::*ERF019* construct, full‐length *ERF019* was directionally cloned into pKANNIBAL (Wesley *et al*., 2001) and then subcloned into the binary vector pART27 (Gleave, 1992). For the CRISPR/Cas9‐based knockouts, two 19‐bp sgRNA oligonulceotides targeting the exon of *ERF019* were inserted in the psgR‐Cas9 vector to create deletion mutants as previously described (Feng *et al*., 2013, 2014). Annealed 19‐bp sgRNA oligomers were inserted into the *Bbs*I site of the psgR‐Cas9 vector. Based on the psgR‐Cas9 vector, the second pATU6‐sgR cassette was amplified by PCR after the insertion of target oligomers and ligated into the *Kpn*I/*Eco*RI site of the above psgR‐Cas9 vector. The cassette was then transferred into the binary vector pCXSN. The generated binary vectors were transformed into *A. tumefaciens* GV3101. *A. tumefaciens* cells carrying 35S::*ERF019* and *sgRNAs/Cas9* constructs were transformed into wild‐type Col‐0 via the floral‐dip method (Zhang *et al*., 2006). 35S::*ERF019* transformants were screened on 1/2 Murashige and Skoog (MS) agar plates containing 50 μg/ml kanamycin. sgRNAs/Cas9 transformants were screened on 1/2 MS agar plates containing 50 µg/ml hygromycin.

### 4.5 *A. tumefaciens* infiltration assays

*A. tumefaciens* GV3101 containing constructs was cultured at 28°C and 200 rpm for approximately 24 hr in Luria Bertani (LB) medium with appropriate antibiotics. The *A. tumefaciens* cells were collected by centrifugation, resuspended in infiltration buffer (10 mM MES, 10 mM MgCl_2_, 0.2 mM acetosyringone, pH 5.6), and adjusted to the appropriate OD_600_ before being infiltrated into 5‐week‐old *N. benthamiana* leaves (the OD_600_ was generally 0.3 for confocal subcellular localization assays and 0.15–0.4 for infection assays). For infection assays, *A. tumefaciens* GV3101 cells carrying constructs were suspended at a concentration of OD_600_ = 0.4, and then inoculated with *P. parasitica* 2 days after infiltration. For co‐expression assays, *A. tumefaciens* GV3101 cells carrying constructs were suspended at an appropriate OD_600_ and mixed before infiltration (for INF1 OD_600_ = 0.15, for Bax OD_600_ = 0.3, for FLAG‐GFP and ERF019 OD_600_ = 0.4). Symptom development was monitored visually from 2 to 6 days after infiltration depending on the cell death activator.

### 4.6 Subcellular localization assays

*A. tumefaciens* GV3101 cell cultures carrying constructs expressing FLAG‐GFP, ERF019‐GFP‐NES, ERF019‐GFP‐nes, ERF019‐GFP or nucleus marker H2B‐mCherry were collected by centrifugation and resuspended in infiltration buffer (10 mM MES, 10 mM MgCl_2_, 0.2 mM acetosyringone, [pH 5.6]) at OD_600_ = 0.4 and then the cells carrying GFP constructs were co‐infiltrated with those expressing the nucleus marker H2B‐mCherry into 5‐week‐old *N. benthamiana* leaves. Three days after infiltration, the *N. benthamiana* leaves were visually inspected under an Olympus FV3000 confocal microscope with excitation wavelengths of 488 nm for GFP and 587 nm for mCherry.

### 4.7 Detection of hydrogen peroxide

Hydrogen peroxide was detected in *Arabidopsis* rosette leaves infiltrated with 1 µM flg22 as described previously (Daudi and O’Brien, 2012).

### 4.8 Oxidative burst measurements

ROS was measured in 30‐day‐old *Arabidopsis* seedlings. In brief, the leaf disks were cut from 30‐day‐old mature leaves with a sharp 5‐mm puncher and were floated in sterile ultrapure water in culture dishes overnight. The next day, the leaf disks were transferred into 96‐well plates and 100 µl Luminol Enhancer (CWBIO, CW0049M) and 100 µl of 20 µg/ml horseradish peroxidase (Aladdin) were added into each cell. Then, 5 µl of 41 µM flg22 was immediately added into each cell to a final concentration of 1 µM. Luminescence was measured using a TECAN Infinite M200 PRO (TECAN); 40 cycles (1 min per cycle) were used for the measurement.

### 4.9 MAPK activity assays

*Arabidopsis* seedlings were grown on vertical 1/2 MS plates at 23°C with 14 hr of light per 24 hr for 12 days and then were transferred into liquid MS and incubated overnight with minimum rotation speed (40 rpm). The next day, the seedlings were treated with 1 µM flg22 and frozen in liquid nitrogen. Total proteins were extracted with glycerol‐Tris‐EDTA‐NaCl buffer (10% glycerol, 25 mM Tris‐HCl [pH 7.5], 1 mM EDTA, 150 mM NaCl, 0.1% Tween 20, 0.1% NP‐40, 2% [wol/vt] PVPP, 0.1 mM DTT, 1 × inhibitor cocktail, 1 × phosphatase inhibitor cocktail 2 and 1 × phosphatase inhibitor cocktail 3). The protein concentration was measured using the Super‐Bradford Protein Assay Kit (CWBIO, CW0013S). Equal amounts of total protein were loaded on a 10% SDS‐PAGE gel. Anti‐phospho‐p44/42 MAPK (Erk1/2) (Thr202/Tyr204) (D13.14.4E) XP rabbit mAb antibody (Cell Signaling Technology) was used to detect the phosphorylation state of MPK3, MPK4, and MPK6.

## AUTHOR CONTRIBUTIONS

W.S., Y.M., and W.L. designed the experiments. W.L., F.D., J.J., X.C., J.L., Q.W., T.L., and Y.M. performed the experiments. W.L., J.J., X.C., Y.M., and W.S. analysed the data. W.L., Y.M., and W.S. wrote the manuscript with contributions from all authors.

## Supporting information


**FIGURE S1**

**FIGURE S2**

**FIGURE S3**

**FIGURE S4**

**FIGURE S5**

**TABLE S1**

## Data Availability

The data that support the findings of this study are available from the corresponding author upon reasonable request.
