# Supplementary figures and images for: The Arabidopsis thaliana gene AtERF019 negatively regulates plant resistance to Phytophthora parasitica by suppressing PAMP‐triggered immunity
Source: Mol Plant Pathol. 2020 Jul 28;21(9):1179–93. doi: 10.1111/mpp.12971 (PMC7411552; doi:10.1111/mpp.12971)

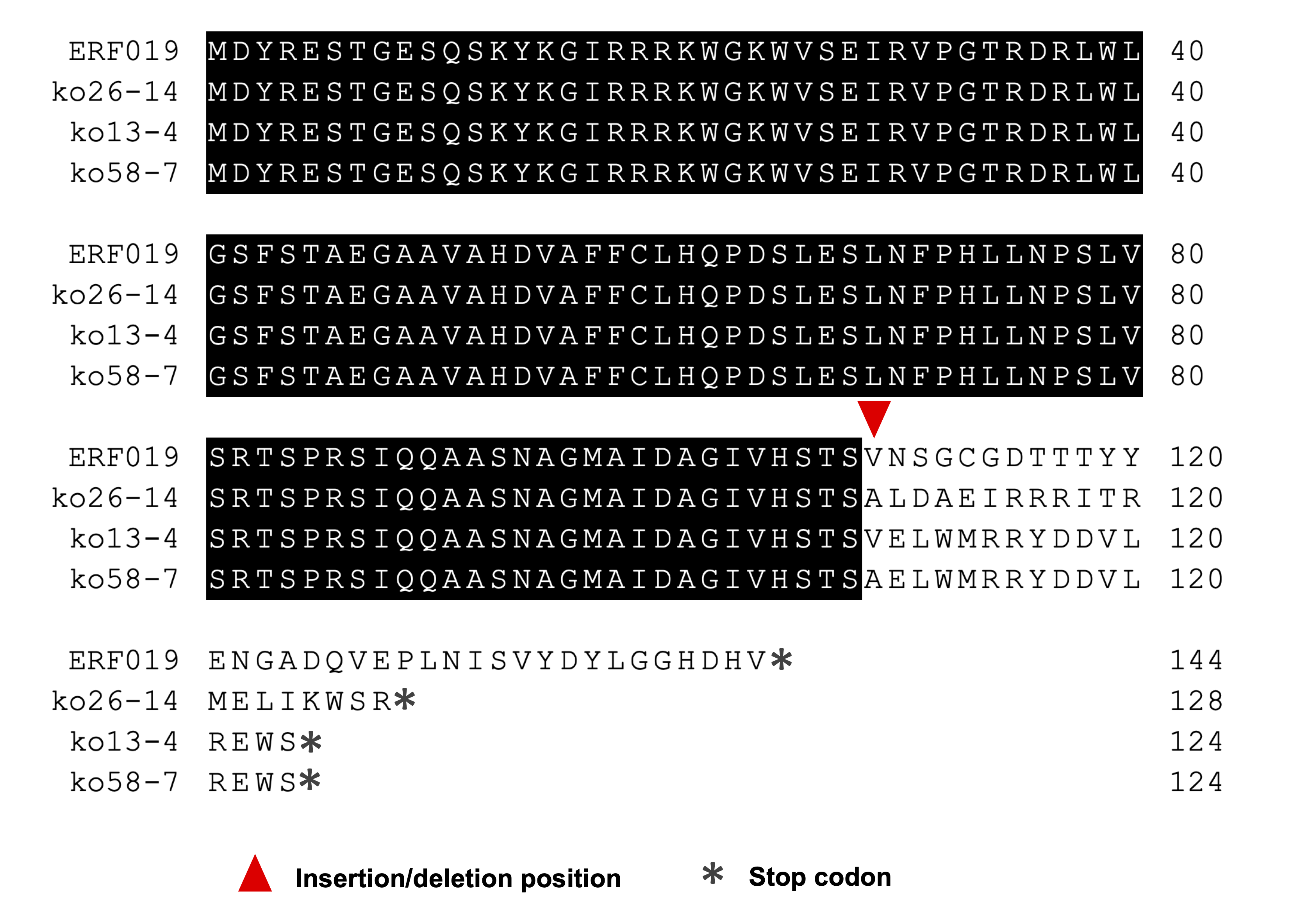

Supplement: Supplementary file 1 — FIGURE S1 [file MPP-21-1179-s001.tiff]

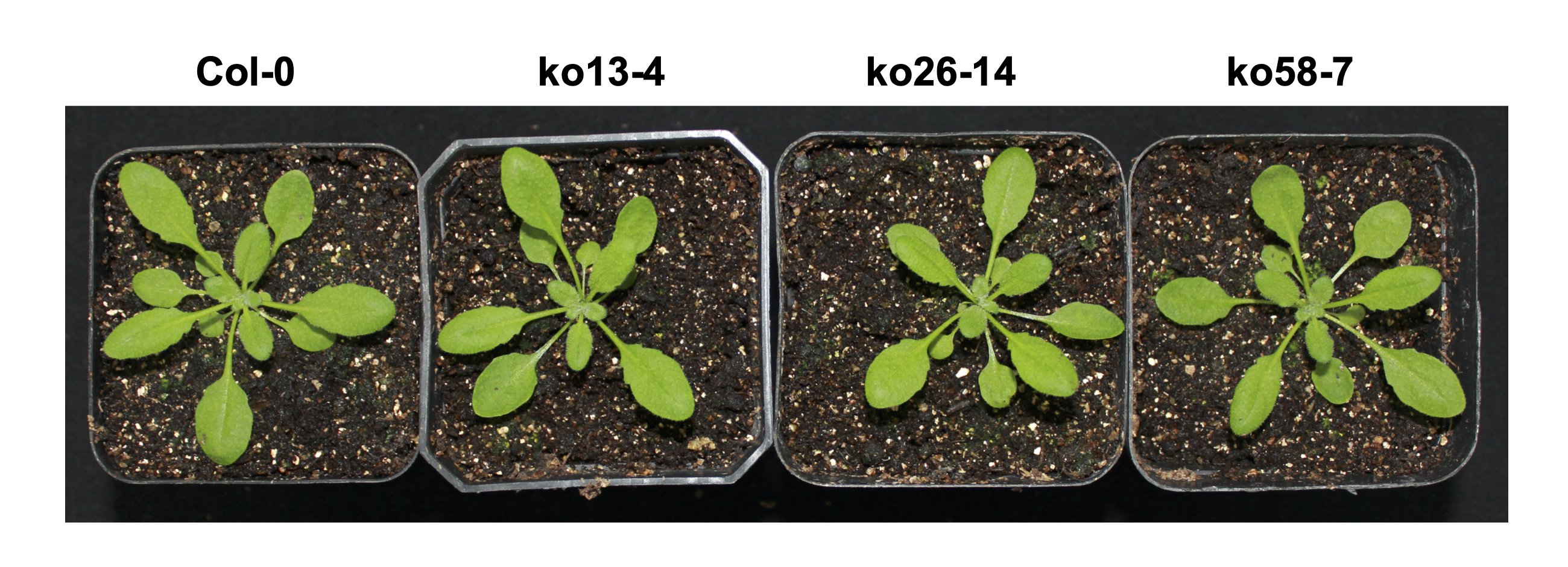

Supplement: Supplementary file 2 — FIGURE S2 [file MPP-21-1179-s002.tiff]

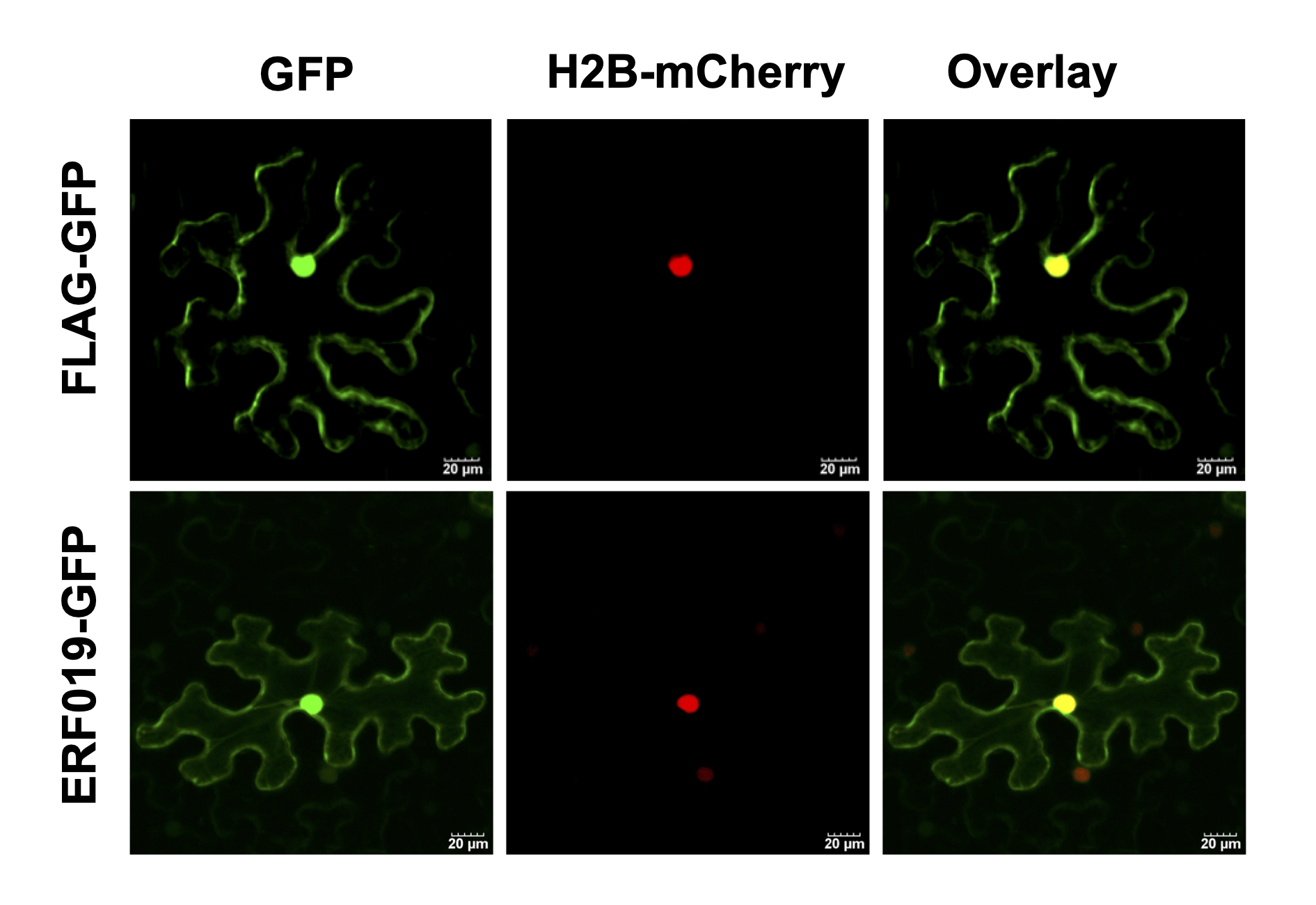

Supplement: Supplementary file 3 — FIGURE S3 [file MPP-21-1179-s003.tiff]

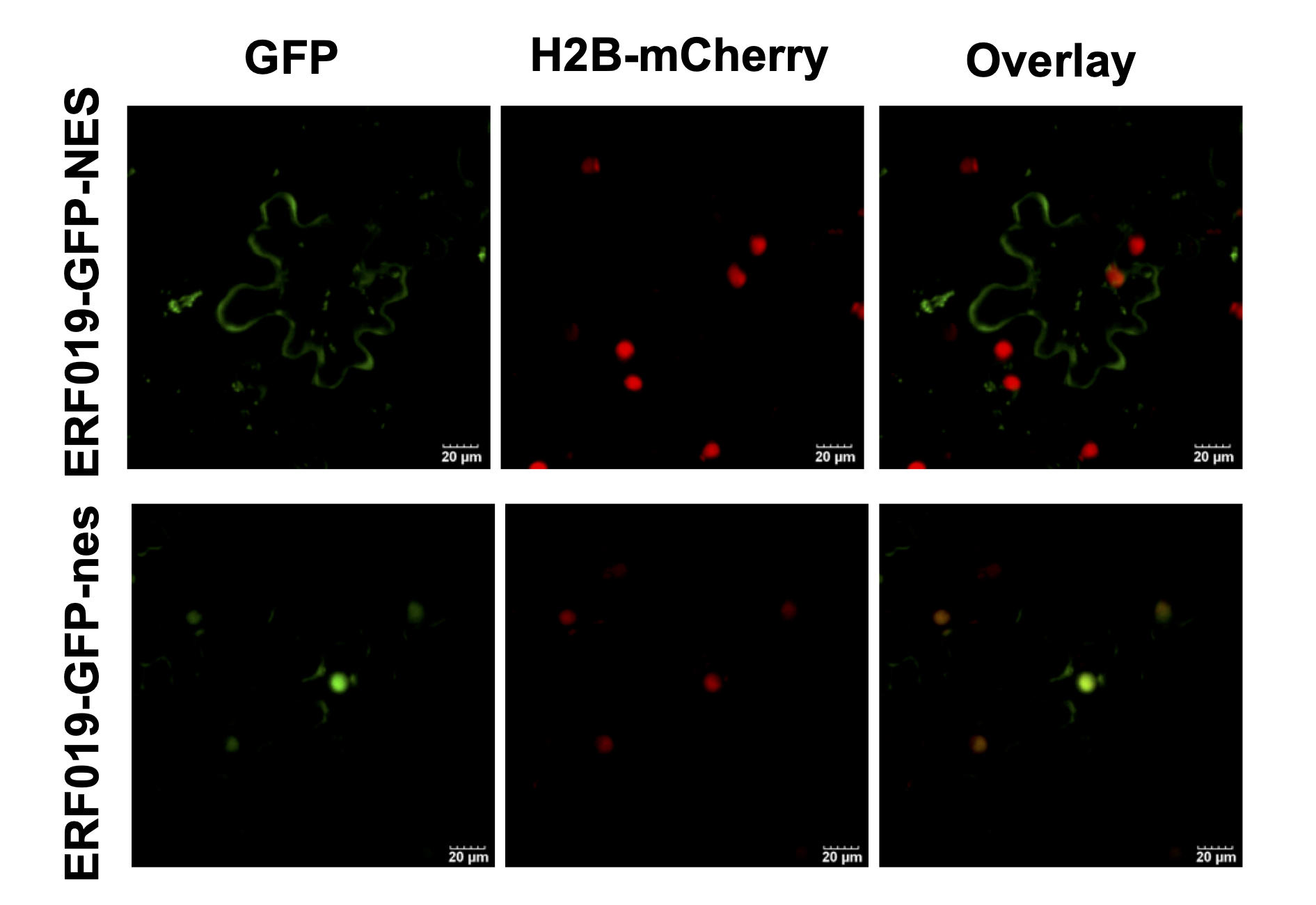

Supplement: Supplementary file 4 — FIGURE S4 [file MPP-21-1179-s004.tiff]

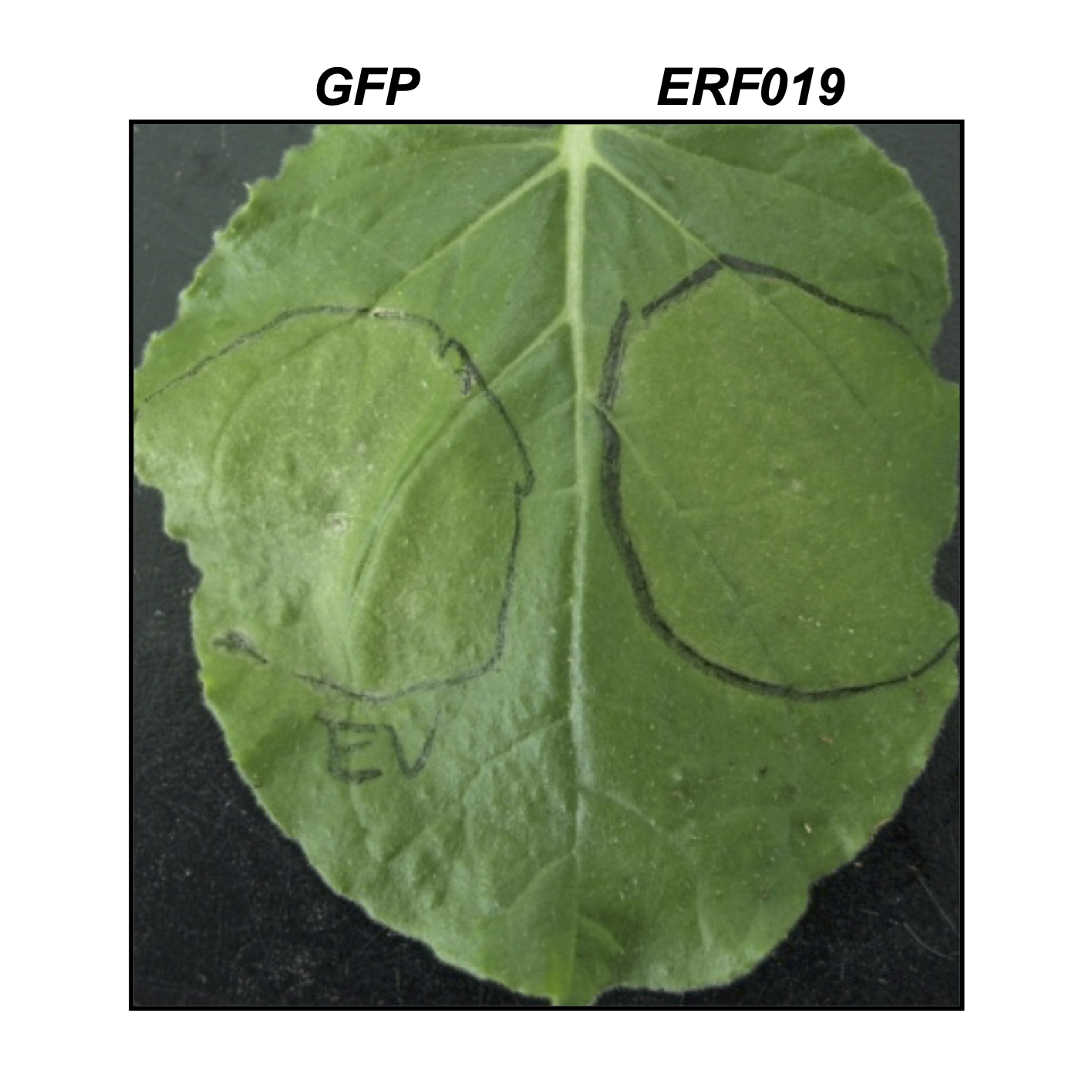

Supplement: Supplementary file 5 — FIGURE S5 [file MPP-21-1179-s005.tiff]
