# Supplementary material for: The Arabidopsis thaliana gene AtERF019 negatively regulates plant resistance to Phytophthora parasitica by suppressing PAMP‐triggered immunity
Source: Mol Plant Pathol. 2020 Jul 28;21(9):1179–93. doi: 10.1111/mpp.12971 (PMC7411552; doi:10.1111/mpp.12971)
Supplement: Supplementary file 6 — TABLE S1 [file MPP-21-1179-s006.docx]

**Table S1 Primers used in this study.**

| purpose | Constructs/Gene name | Primer name | Sequence (5' - 3') |
| --- | --- | --- | --- |
| Overexpression | *pART27*::*ERF019* | ERF019-F | CCGCTCGAGATGGATTACAGAGAATCCACCG |
|  |  | ERF019-R | GCTCTAGA TCAAACGTGATCGTGGCCG |
| CRISPR/Cas9 editing | *pCXSN*::*ERF019* | ERF-sgRNA-F1 | GATTGCCGCATCCAGAGTTCACGC |
|  |  | ERF-sgRNA-R1 | AAACGCGTGAACTCTGGATGCGGC |
|  |  | ERF-sgRNA-F2 | GATTGCTGGTACTGTGGACGATTC |
|  |  | ERF-sgRNA-R2 | AAACGAATCGTCCACAGTACCAGC |
| Overexpression and subcellular localization | 35S::*ERF019-GFP* | ERF019-F | CCGCTCGAG ATGGATTACAGAGAATCCACCG |
|  |  | ERFGFP-R | CTCCTCGCCCTTGCTCACCATAACGTGATCGTGGCCG |
|  |  | C-GFP-F | ATGGTGAGCAAGGGCGAGGA |
|  |  | C-GFP-R | GCTCTAGA TTACTTGTACAGCTCGTCCATGC |
|  | 35S::*ERF019-GFP-NES* | NES-R | GGACTAGTTTAGATATCCAACCCAGCCAATT |
|  | 35S::*ERF019-GFP-nes* | nes-R | GGACTAGTTTACGCATCAGCACCAGCCGCTT |
| qRT-PCR primers | ERF019 (AT1G22810) | QERF019-F | GCACACGACGTTGCTTTCTTC |
|  |  | QERF019-R | GTCGTCGTATCTCCGCATCC |
|  | UBC9 (AT4G27960) | QAtUBC9-F | CATCGGATAGCCCTTATTCTG |
|  |  | QAtUBC9-R | TGGAACACCTTCGTCCTAAAA |
|  | FRK1 (*AT2G19190*) | QFRK1-F | AGCGGTCAGATTTCAACAGT |
|  |  | QFRK1-R | AAGACTATAAACATCACTCT |
|  | LOX2 (*AT3G45140*) | QLOX2-F | TGCACGCCAAAGTCTTGTCA |
|  |  | QLOX2-R | TCAGCCAACCCCCTTTTGA |
|  | PR1 (*AT2G14610*) | QPR1-F | GCTAACTACAACTACGCTGCGAA |
|  |  | QPR1-R | TCTCGTTCACATAATTCCCACGA |
|  | PDF1.2 (*AT5G44420*) | QPDF1.2-F | CCATCATCACCCTTATCTTCGC |
|  |  | QPDF1.2-R | CCATGTTTGGCTCCTTCAAGGTT |
|  | VSP2 (*AT5G2477*0） | QVSP2-F | TTGGCAATATCGGAGATCAAT |
|  |  | QVSP2-R | GGGACAATGCGATGAAGATAG |
|  | ICS1 (AT1G74710) | QICS1-F | GCCGTCTCTGAACTCAAATCTCAA |
|  |  | QICS1-R | CTCCAATCGTCATGAGAGGAA |
|  | PAL1 (AT2G37040) | QPAL1-F | TGTAGCGCAACGTACC |
|  |  | QPAL1-R | GTTCGGGATAGCCGATG |
|  | ACS2 (AT1G01480) | QACS2-F | GGATGGTTTAGGATTTGCTTTG |
|  |  | QACS2-R | GCACTCTTGTTCTGGATTACCTG |
|  | ACS6 (AT4G11180) | QACS6-F | GTTCCAACCCCTTATTATCC |
|  |  | QACS6-R | CCGTAATCTTGAACCCATTA |
|  | EIN2 (AT5G03280) | QEIN2-F | CCTTGTCACTAATGGAGCAGG |
|  |  | QEIN2-R | CACGATGAAGCCAAGCG |
|  | ERF6 (AT4G17490) | QERF6-F | GAAAACCGCCGTTGAAGATC |
|  |  | QERF6-R | CGGTTGCGAATTGAATCCA |
| Biomass primers | AtUBC9 | AtUBC9-Fg | TTCATTGGCAGGCCACTAT |
|  |  | AtUBC9-Rg | CTTAGGAGGCTTAAATGGGTAA |
|  | PpUBC9 | PpUBC-F | CCACTTAGAGCACGCTAGGA |
|  |  | PpUBC-R | TACCGACTGTCCTTCGTTCA |
